# Rethinking surgical intervention in the multidisciplinary comprehensive management of craniocerebral metastasis in multiple myeloma

**DOI:** 10.1097/MD.0000000000047258

**Published:** 2026-01-23

**Authors:** Junjie Shen, Yong Cai, Hao Ouyang, Xingming Zhong

**Affiliations:** a Department of Neurosurgery, Huzhou First People's Hospital, The First Affiliated Hospital of Huzhou University, Huzhou, Zhejiang, P.R. China.

**Keywords:** intracranial metastasis, multiple myeloma, prognosis, surgical intervention

## Abstract

**Rationale:**

Multiple myeloma (MM) is a common hematologic malignancy characterized by widespread systemic involvement. Radiation therapy is the 1st-line treatment for MM patients with brain metastases in clinical practice, whereas surgical intervention is only sporadically reported in special cases.

**Patient concerns:**

A MM patient receiving conventional chemotherapy presented with progressive motor dysfunction of the right limb. Imaging examinations revealed a space-occupying lesion in the left parietal lobe.

**Diagnoses:**

Following multidisciplinary consultation involving hematology and neurosurgery departments, the patient was diagnosed with central nervous system multiple myeloma (CNS-MM).

**Interventions:**

Prompt symptomatic treatments, including intracranial pressure reduction and antiepileptic therapy, were initiated, but the patient’s muscle strength continued to deteriorate to grade II. A left parietal lobe brain tumor resection was then performed after comprehensive evaluation.

**Outcomes:**

Postoperatively, the patient’s muscle strength recovered rapidly, and no tumor recurrence was noted during follow-up. However, the patient eventually died of uncontrolled pulmonary *Staphylococcus aureus* infection.

**Lessons:**

CNS-MM is associated with a poor prognosis, and the current treatment strategies remain suboptimal. Surgical intervention may be a viable option for patients with rapidly progressive CNS-MM. This case comprehensively analyzes the potential benefits of surgical treatment for specific CNS-MM patients, based on existing treatment protocols and relevant case reports.

## 1. Introduction

Multiple myeloma (MM) is a plasma cell malignancy characterized by clonal proliferation, with lesions primarily confined to the bone marrow. However, in a small subset of patients, the disease can involve extramedullary sites, with an incidence of approximately 5%. The most commonly affected sites include the liver, spleen, and lymph nodes. Among these extramedullary manifestations, central nervous system (CNS) involvement is exceptionally rare, with an incidence of <1%.^[1,2]^ This retrospective study discusses the clinical data of a patient treated at the Neurosurgery Department of the First People’s Hospital of Huzhou, Zhejiang Province, who developed intracranial metastatic disease during chemotherapy, along with a review of the literature. This case has several notable characteristics: no craniocerebral metastasis was confirmed and no neurological related lesions were present at the initial diagnosis of MM; after the occurrence of intracranial metastasis, the patient was mainly treated with neurosurgical surgery, and the intracranial mass effect subsided rapidly after the resection of the mass; and we completed a thorough follow-up of the patient and found no signs of recurrence of the intracranial mass.

## 2. Case report

Patient information: a 59-year-old female presented with “lumbar discomfort” at the 72nd Group Army Hospital in Huzhou, China, in 2022. Laboratory results revealed hemoglobin of 81 g/L, total protein of 104.3 g/L, albumin of 27.4 g/L, and serum protein electrophoresis indicated a prominent band in the gamma region. On June 7, 2021, the patient was admitted to the Hematology Department of the First People’s Hospital of Huzhou, where a bone marrow biopsy led to a preliminary diagnosis of “multiple myeloma” (IgA-K type, Durie-Salmon stage IIIA, International Staging System stage III, FGFR3 positive) according to the 2015 revised Chinese guidelines for MM. Treatment course: starting on June 15, 2021, the patient underwent standard chemotherapy. By December 2023, due to inadequate disease control, her treatment regimen was altered multiple times, including VRD, PCD, IRD, KD, and KPD protocols. During treatment, the patient experienced complications such as herpes zoster, urinary calculi, bone marrow suppression, and urinary tract infections. In December 2023, she developed progressive right-sided limb weakness, leading to further evaluations that indicated a space-occupying lesion in the brain, necessitating transfer to neurosurgery for surgical intervention. Based on the CT and MRI findings, meningioma was initially considered, and cranial invasion by MM could not be ruled out. During hospitalization, the patient completed preoperative preparations for meningioma surgery, was given immunoglobulin and antiepileptic drugs, and further CTV examination was performed due to the location of the space-occupying lesion near the sagittal sinus. Throughout conservative management, the patient remained conscious (glasgow coma scale = 15), although her right-sided muscle strength declined from grade V to grade II, indicating surgical intervention was warranted.

**Figure 1.**
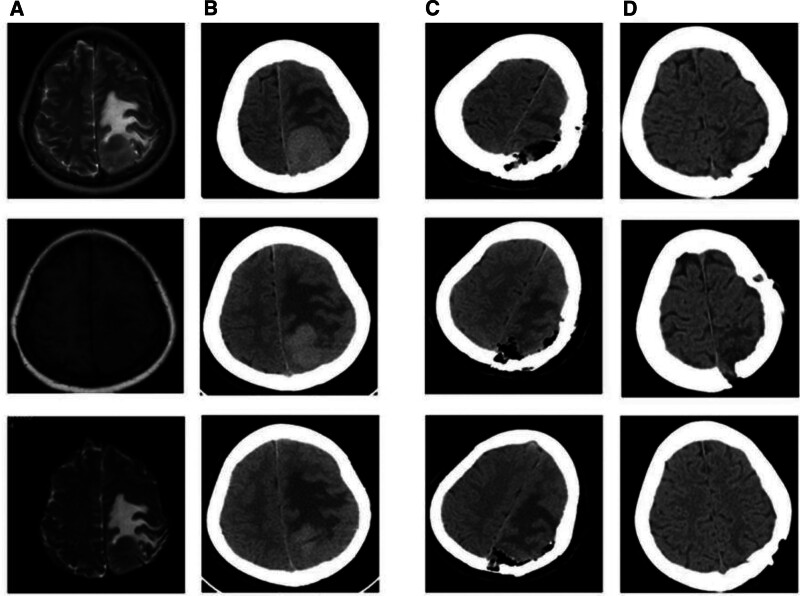
The imaging studies conducted for the patient include: (A) preoperative MRI on December 28, 2023. (B) Preoperative CT on January 2, 2024. (C) Postoperative CT on January 3, 2024. (D) Postoperative CT on March 14, 2024.

Imaging studies: (Preoperative cranial MRI on December 28, 2023) A mass-like lesion with a size of approximately 31 mm × 29 mm was observed in the left parietal region (parasagittal sinus). It showed slightly low signal intensity on T1-weighted imaging, isointense signal on T2-weighted imaging with a surrounding edema zone, and slightly high signal intensity on diffusion-weighted imaging, which might be related to the relatively high cell density. After contrast enhancement, the lesion showed obvious enhancement with a “dural tail sign,” suggesting a meningeal origin. Patchy long T2 signal shadows were seen around, and the left ventricle was compressed (Fig. 1A). (Preoperative cranial CT on January 2, 2024) A round-like slightly high-density shadow with a size of approximately 38 × 30 mm was found in the left parietal region, with obvious local brain tissue compression, a significant surrounding edema zone, and compression of part of the left ventricle (Fig. 1B). (Preoperative CTV on December 29, 2023) Angiography showed that the lesion was adjacent to the superior sagittal sinus, causing venous compression and local reflux obstruction. Combined with the patient’s examination and clinical manifestations, it was evaluated that the tumor had a risk of further enlargement and compression.

Laboratory and other investigations: blood tests revealed decreased total protein and prealbumin levels, elevated β2-microglobulin, lipoprotein, and immunoglobulin A, along with elevated LDH. Bone marrow cytology showed markedly reduced nucleated cell proliferation, with a granulocyte-to-erythroid ratio of 3.0 and nearly absent erythroid precursors. FISH analysis showed 80% positivity for IGH/FGFR3, 90% for p53 deletion, and 35% for 1q21 amplification. Chromosomal analysis revealed abnormalities consistent with MM.

**Figure 2.**
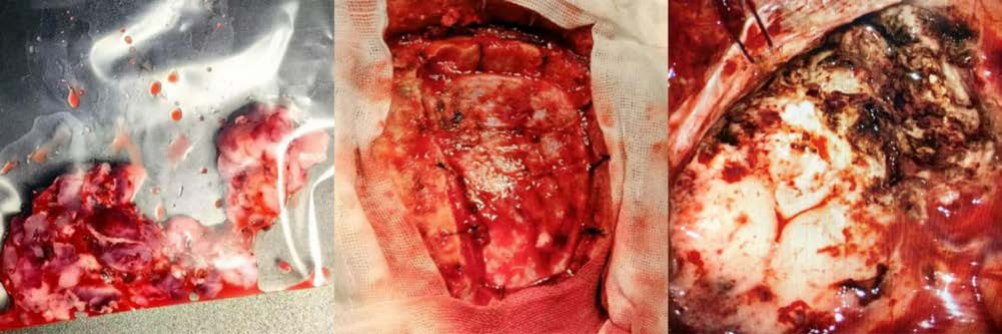
During the surgery, elevated intracranial pressure was observed. The tumor appeared fleshy, with rich blood supply, and infiltrating the sagittal sinus. Its texture was relatively firm, and the boundaries with the surrounding brain tissue were unclear.

Surgical intervention: the patient was placed in the prone position during the operation. A U-shaped incision was made in the left parietal lobe, and the skin, subcutaneous tissue, and galea aponeurotica were incised layer by layer. After separating the periosteum and exposing the skull, the skull was routed and the bone flap was removed according to the range of the incision. It was observed that the dura mater had high tension and a full shape, and no signs of tumor invasion were found in the epidural space and the inner table of the skull. Subsequently, the dura mater was incised along the predetermined range, exposing a grayish-white, fish-flesh-like mass under the dura mater, which was tough in texture, prone to bleeding, had a clear boundary with the surrounding dura mater, was close to but not invading the sagittal sinus, and adhered to the brain parenchyma; the mass extended deep into the falx cerebri, with a clear boundary from the falx cerebri and no invasion. During the operation, the separation was first performed step by step along the boundary between the mass and the dura mater. For the adherent reflux veins, low-power electrocoagulation was used to carefully handle the branches to avoid damaging the main trunk. For the part adhering to the falx cerebri, the tissues beside the falx were incised along the edge of the tumor for gradual deep separation and resection. Under the premise of protecting the surrounding normal brain tissue, the tumor was resected as completely as possible, and the resected mass was immediately sent for rapid pathological examination (Fig. 2). Finally, after confirming that there was no residual tumor and active bleeding, autologous muscle fascia was used for dural repair to suture tightly to prevent cerebrospinal fluid leakage. The bone flap was reset and fixed, and then the galea aponeurotica, subcutaneous tissue, and skin were sutured layer by layer to complete the operation.

**Figure 3.**
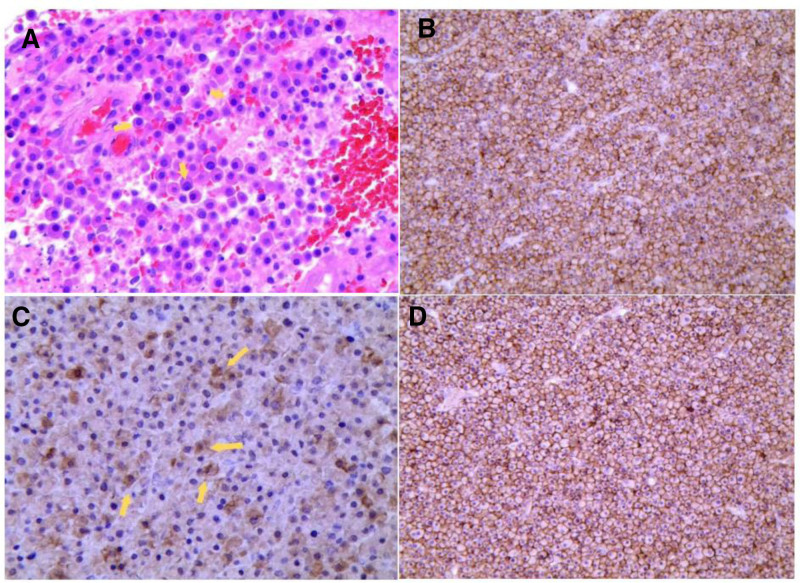
Specimen analysis results. (A) HE100 staining shows abundant plasma cells with nuclear atypia throughout the field. (B) CD38 is membrane positive. (C) CD79a is cytoplasm positive. (D) CD56 is membrane positive.

Pathological analysis: microscopic examination revealed abundant plasma cells with red-stained nuclei. Immunohistochemistry indicated positive CD38, CD56, and MUM1, significant KI-67 proliferation, and positive CD79a. Genetic testing identified 2 potentially disease-associated mutations, IGLLS and SETD1B (Fig. 3).

Postoperative prognosis and outcome: immediately after the operation, the patient underwent a CT examination, which showed that the tumor was completely resected, and there was accumulated blood and patchy edema in the surgical area (Fig. 1C). Subsequently, the patient was transferred to the ICU for further treatment. On the first day after the operation, the patient regained stable spontaneous breathing, the endotracheal tube was removed, the consciousness became clear, and the bilateral light reflexes were normal. On the second day after the operation, the patient was evaluated and transferred to the general ward for continued treatment. During this period, conventional treatments such as blood pressure lowering, antiepileptic, antiviral and rehabilitation therapies were given. The patient was discharged 24 days after the operation. At the time of discharge, the glasgow coma scale score was 15, the bilateral pupils were equal in size with sensitive light reflexes, the muscle strength of the left side was normal, and the muscle strength of the right side was grade IV.

the patient was transferred to the Department of Hematology for further treatment. Due to the presence of extramedullary disease and the pathological report indicating CD38 membrane positivity, 2 targeted drugs, daratumumab and selinexor, were additionally administered on the basis of pomalidomide treatment. During this period, the muscle strength of the patient’s limbs was normal, and reexaminations of cranial CT showed no signs of tumor recurrence (Fig. 1D). During the subsequent hospitalization, the patient developed a *Staphylococcus aureus* infection. Despite prompt treatment, unfortunately, due to factors such as underlying diseases and the patient’s decreased immune function, the infection could not be effectively controlled. The patient passed away after receiving 4 months of drug treatment.

## 3. Discussion on MM and CNS involvement

MM, a common hematological malignancy, accounts for approximately 1.8% of all malignant tumors and up to 10% of hematological-related tumors, with an increasing trend year by year.^[1]^ MM is more prevalent in males and is often accompanied by symptoms such as bone damage, anemia, infection, renal insufficiency, immune dysfunction, and neurological damage. The timing of CNS-MM in patients with MM varies. Some patients present with CNS-MM at the time of initial MM diagnosis, while more develop it within 2 to 3 years after diagnosis. CNS-MM predominantly affects middle-aged and elderly populations. Its key features include plasma cell infiltration of the CNS, meninges, or cerebrospinal fluid. Patients with CNS-MM typically have an extremely poor prognosis, with a median survival of usually <6 months.^[3]^ CNS-MM is mainly caused by direct spread from adjacent bones and hematogenous dissemination, usually occurring in the meninges, with the least involvement of the brain parenchyma. Most of these diseases occur during recurrence or progression of the disease, and cases with neurological abnormalities as the initial diagnosis (i.e., primary CNS-MM) account for only 20%.^[4]^ In addition, due to the differences in the location of intracranial invasion by plasmacytoma and the specificity of MM itself, patients often have different clinical manifestations, mostly including progressive decline in muscle strength, limb numbness, hearing loss, epileptic seizures, and bleeding tendency; there have even been reports of MM patients with intracranial hemorrhage as the first symptom, even if the patients had never shown symptoms related to MM before, which makes us think about the possibility of MM when diagnosing patients with intracranial hemorrhage.^[5]^

In this case, the patient had no symptoms of neurological involvement at the time of MM diagnosis, but had some high-risk factors for the occurrence of CNS-MM, such as age < 65 years, IgA type, and more than 2 osteolytic lesions, and developed intracranial mass effect during chemotherapy.^[6]^ In addition to the existing high-risk factors of this patient, the occurrence of CNS-MM is also related to plasmablastic morphology, cytogenetics, etc, but due to the patient’s economic reasons, we did not conduct further testing. Cerebrospinal fluid testing is also a strong evidence for the diagnosis of CNS-MM. Almost all patients can find atypical plasma cells or anaplastic plasma cells in the cerebrospinal fluid, and CD38 and CD138 are also expressed in cell flow cytometry.^[7]^ However, because the examination requires a large amount of cerebrospinal fluid and may need multiple punctures, the patient refused this examination, so we lost this part of the data. Nevertheless, the analysis of postoperative pathological specimens also found positive expression of CD38 and CD56. Similar to the patient in this case, the cause of CNS-MM during treatment has not been determined, but it is certain that new treatment methods and early diagnosis of the disease have improved the survival period of patients, thereby increasing the incidence of CNS-MM. At present, no studies have reported that the use of new chemotherapy drugs will lead to an increase in the incidence of CNS-MM. Only a few cases have mentioned abnormal changes in the incidence of CNS-MM during thalidomide treatment, which may be related to thalidomide-induced downregulation of CXCL12 (SDF-1a)/CXCR4, thereby weakening the interaction between plasma cells and surrounding bone marrow stroma.^[8,9]^ Some researchers have also put forward hypotheses: CNS-MM may be the result of the survival and selection of some plasma cell subclones outside the medullary cavity after successful treatment with chemotherapy drugs or autologous stem cell transplantation. New drugs have weak penetration of the blood–brain barrier, so they cannot inhibit the proliferation of plasma cells in the intracranial region,^[4]^ but more experiments are needed to confirm this.

Currently, the treatment of CNS-MM still faces significant challenges, and various research protocols have failed to achieve satisfactory results. The existence of the blood–brain barrier leads to insufficient efficacy and limited choices of drugs for treating CNS-MM. Most drugs currently used for the treatment of CNS-MM have limitations. Thalidomide is a commonly used immunomodulatory drug in clinical practice. Although it has a strong ability to penetrate the blood–brain barrier and is mostly used in combination therapy, its efficacy exerts slowly, resulting in poor therapeutic effects on rapidly progressive CNS-MM.^[10,11]^ Pomalidomide also possesses favorable blood–brain barrier penetration ability and exhibits good efficacy in the treatment of CNS-MM. However, current research on its application in CNS-MM is relatively scarce, and only some case reports have mentioned the therapeutic effects of its intrathecal injection.^[12,13]^ In the treatment of CNS-MM, the relatively new agent bortezomib is mostly used in combination with thalidomide and lenalidomide. However, it does not show significant advantages over other drugs in terms of patients’ survival rate and remission rate, which is presumably related to its weak ability to penetrate the blood–brain barrier.^[14,15]^ In other CNS-MM treatment regimens, such as intrathecal injection of methotrexate or cytarabine combined with hydrocortisone, their efficacy is currently unclear because they are usually used in combination with other systemic chemotherapy, and there are case reports that single-agent use failed to improve patient survival.^[16]^ Melphalan is often used in the conditioning regimen for autologous hematopoietic stem cell transplantation and has a good ability to penetrate the blood–brain barrier. Some case reports show that it can improve patients’ survival time from 1.5 to 25 months. Although no statistical difference is achieved, it still has certain positive significance.^[4,17]^

In addition, since B-cell maturation antigen is highly expressed on the surface of myeloma cells, B-cell maturation antigen therapy has emerged as one of the hotspots in recent years. Globally, ciltacabtagene autoleucel and eculizumab (the 2 agents in CAR-T therapy) have been successively approved. Currently, there are not only case reports on their application in the treatment of CNS-MM, but also their remission rates show favorable performance. Meanwhile.^[18,19]^ although bispecific antibody (BsAb) therapy has potential toxic risks, studies have applied it to the treatment of CNS-MM, and no adverse reactions have been observed.^[20–22]^

Neurosurgical resection is not the first-line treatment for intracranial invasion of MM, and its efficacy has not been determined in clinical practice. Since drug therapy can improve patients’ symptoms to a certain extent, and most patients prefer drug therapy, even when CNS-MM patients have mild mass effect, drugs and radiotherapy are still the majority of treatment options. However, when patients have a series of symptoms such as progressive decline in muscle strength and increased intracranial pressure, or when the space-occupying lesion is located in areas such as the optic chiasm where drug treatment has poor effect, we may have to perform surgical intervention to resect the tumor to improve the progression of the patient’s condition and ensure the patient’s life. At present, some articles have reported the effect of surgical treatment for craniocerebral metastasis of MM. In a case reported by Crowley RW,^[23]^ although the patient was in the remission period of MM and there was no evidence of myeloma recurrence in the examination, he still developed symptoms such as progressive right limb weakness and drowsiness. At the same time, CT showed left temporal hemorrhage with significant mass effect, and then surgery was performed to completely remove the hematoma and mass. The patient’s muscle strength recovered rapidly after the operation. Although dysphagia occurred, it was completely relieved within 1 year of rehabilitation treatment. Subsequently, the patient received conventional drug therapy and radiotherapy after the operation. Multiple reexaminations of CT showed no tumor recurrence, and the survival period has exceeded 4 years. In a case provided by Onodera K,^[24]^ a patient with MM developed disturbance of consciousness and right hemiplegia during chemotherapy. MRI showed massive cerebral hemorrhage and significant mass effect in the left temporal region, which were life-threatening. The patient immediately received surgical treatment. After the operation, the patient’s hemiplegia and aphasia disappeared, and further antitumor treatment was received 2 weeks after the operation. At discharge, there were no neurological deficits and the CR state lasted for 6 months. In the case provided by Prakash A,^[25]^ the patient had multiple syncopes 16 months after the diagnosis of MM. MRI showed a left frontal space-occupying lesion and midline shift, but there were no abnormalities in other neurological and hematological examinations. Later, the patient underwent stereotactic craniotomy. During the 1-year follow-up, although there was evidence of active disease, there was no recurrence of syncope. In addition, some patients with CNS-MM have intracranial space-occupying lesions as the first symptom, but this situation is very rare and lacks clear imaging diagnostic basis, so patients often receive surgery with the diagnosis of intracranial space-occupying lesions. At present, some articles have also reported the prognosis of such patients. A patient with “meningioma” presenting with language expression disorder and right limb weakness was pathologically diagnosed as MM after surgical treatment. After receiving subsequent radiotherapy and chemotherapy, the neurological symptoms completely disappeared, and there was no recurrence or progression during the subsequent 3-month follow-up.^[26]^ However, there are also some failed cases. In an early report by Mäntylä R^[27]^ in 1995, a patient had a 5 cm high-density space-occupying lesion found by CT and the condition deteriorated rapidly. After the occurrence of a series of clinical symptoms such as increased intracranial pressure, aphasia and hemiplegia, surgical resection was performed quickly. After the operation, the patient’s hemiplegia and mental state improved slightly, but unfortunately, cerebral hemorrhage and cerebellar tonsillar herniation occurred during the subsequent visit to the local hospital, which directly led to the patient’s death. However, limited by the medical conditions at that time, coupled with the discovery of incompletely resected tumors in the autopsy, these may be related to the patient’s death to a certain extent, so the current surgical treatment effect cannot be completely denied.

In the aforementioned cases, when MM metastasizes to the intracranial region, leading to a series of symptoms such as severe intracranial mass effect and cerebral hemorrhage, neurosurgical treatment can rapidly alleviate issues such as language dysfunction, muscle weakness, and syncope. Additionally, despite the use of craniotomy (as in the case of the patient in this report), most relevant studies indicate that surgery does not result in severe neurological damage. In terms of prognosis and survival, all patients in the aforementioned cases survived for more than 6 months. The potential risks and adverse prognoses associated with craniotomy cannot be ignored, but with advancements in medical technology, such drawbacks are continuously being mitigated. In this case, the patient had received various immunotherapeutic drugs and proteasome inhibitors before the development of the intracranial mass lesion, which may be associated with the occurrence of CNS-MM. Furthermore, the patient’s imaging findings did not directly support a diagnosis of intracranial metastasis of MM; instead, they were more consistent with meningioma. This diagnostic bias may have affected the timeliness and accuracy of early treatment decisions. If the patient’s CNS-MM had been clearly diagnosed at an early stage and the condition was stable, more treatment options might have been available (such as stereotactic radiotherapy). However, given the tendency of the patient’s mass to enlarge, the rapid progression of the disease, and the rapid decline in muscle strength, coupled with the patient’s urgent desire to alleviate symptoms to avoid life-threatening complications, clinical practice necessitated early surgical intervention to address the emergency. In terms of surgical outcomes, the patient’s muscle strength recovered rapidly after surgery, with no surgery-related adverse reactions. Subsequent follow-up examinations also showed no signs of intracranial tumor recurrence. Moreover, the tumor specimens obtained directly through surgery, after pathological analysis, provided important basis for the precise selection of subsequent drug treatments. In addition to routine pathological examinations, we also performed genetic testing on the tumor, but no clear tumor-related mutations were identified, only 2 potentially related mutations were found, so we could not administer targeted therapy based on the patient’s genetic mutations. For the postoperative drug treatment regimen, after comprehensive evaluation, weekly daratumumab combined with selinexor was chosen. It should be noted, however, that these 2 drugs have hematological toxicity and may exert certain effects on the patient’s immune system. It is important to note that the hematological toxicity induced by dara-pom therapy impairs patients’ immune function. Additionally, the patient’s prior anti-infective treatment for recurrent urinary tract infections led to decreased antibiotic sensitivity. These 2 factors collectively increased the risk of infection and treatment difficulty during subsequent management. Four months after surgery, the patient developed unexplained fever. After excluding intracranial infection and osteomyelitis through examinations, the infection was confirmed to be pulmonary *S aureus*. Unfortunately, due to the patient’s immunocompromise and poor response to antibiotic therapy, the infection remained uncontrolled, and the patient ultimately succumbed to the illness. A review of clinical cases shows that while surgery rapidly relieved patients’ symptoms, severe infections from the disease itself and subsequent treatment remind us to prioritize immune intervention for such patients. In CNS-MM, the disease impairs the immune system, and drug therapy further suppresses immunity – leaving patients prone to immunocompromise and infection. Thus, monitoring immune status and timely prevention are critical. Beyond treating the primary disease to reduce immune damage, strategies include: predicting immunity via T-cell surface receptor protein (e.g., PD-1 and CD28) testing, using immunoglobulin to regulate immunity, and prophylactic anti-infective therapy based on results. For persistent severe lymphopenia during treatment, balance “antitumor efficacy” and “immune protection,” adjusting drugs if needed.

## 4. Summary

CNS-MM is extremely rare. Compared with other intracranial tumors, it lacks specific clinical manifestations; given its extremely poor prognosis, risk assessment and subsequent treatment for CNS-MM are crucial. For patients diagnosed with MM, if neurological symptoms occur, comprehensive and detailed examinations should be conducted to rule out CNS-MM. Currently, although various treatment regimens have been used clinically for CNS-MM, their efficacy remains unclear. In addition, patients usually have a short survival time, so close and thorough follow-up monitoring of such cases is essential. Meanwhile, reporting these rare cases is of great value for future related research, which may provide a reference for optimizing clinical diagnosis and treatment strategies.

## Author contributions

**Resources:** Xingming Zhong.

**Supervision:** Yong Cai.

**Writing – original draft:** Junjie Shen.

**Writing – review & editing:** Hao Ouyang.
